# Comprehensive characterisation of age-related changes in cell subpopulations and tissue structural properties in secondary lymphoid organs

**DOI:** 10.1038/s41419-025-08007-y

**Published:** 2025-10-06

**Authors:** Yuxin Deng, Xin He, Juzheng Peng, Yuxi Pan, Yusheng Luo, Yueheng Ruan, Jianfeng Hou, Bangxue Jiang, Xiangyu Li, Xiaomei Liang, Jiayuan Huang, Jiancheng Wang

**Affiliations:** 1 Scientific Research Center, The Seventh Affiliated Hospital of Sun Yat-sen University, Shenzhen, Guangdong China; 2 Zhongshan School of Medicine, Sun Yat-sen University, Guangzhou, Guangdong China; 3 Department of Hematology, The Seventh Affiliated Hospital of Sun Yat-sen University, Shenzhen, Guangdong China; 4 Department of Oncology, The Seventh Affiliated Hospital of Sun Yat-sen University, Shenzhen, Guangdong China; 5 Digestive Diseases Center, Guangdong Provincial Key Laboratory of Digestive Cancer Research, The Seventh Affiliated Hospital of Sun Yat-sen University, Shenzhen, Guangdong China; 6 School of Medicine, Shenzhen Campus of Sun Yat-sen University, Shenzhen, Guangdong China; 7 Department of Orthopedic Surgery, The Seventh Affiliated Hospital of Sun Yat-sen University, Shenzhen, Guangdong China

**Keywords:** Cell biology, Immunopathogenesis

## Abstract

Population aging is an escalating global phenomenon, wherein age-related alterations in the human immune system exacerbate the susceptibility to diseases including infections and autoimmune disorders. Secondary lymphoid organs (SLOs) are key locations for the execution of immunological responses by mature immune cells; however, age-related changes in SLOs remain relatively understudied. To address this gap, this study employed comprehensive approaches including single-cell RNA sequencing (scRNA-seq) data analysis, immunofluorescence staining, flow cytometry, and morphological analysis, to clarify the age-related alterations in SLOs in mice. The results demonstrated that aging caused senescent immune cells to accumulate and subpopulations to reorganize, with a decrease in the proportion of naïve T cells, whilst an increase in regulatory T (Treg) cells, cytotoxic T lymphocytes (CTLs), and exhausted T (Tex) cells. Notably, CD4^+^ and CD8^+^ T cells exhibited distinct senescence patterns in Peyer’s patches, suggesting tissue-specific responses to aging, which may arise from differential exposure to gut microbiota. In addition to the alterations in immune cell populations, we also identified increased stromal cell senescence and altered distributions of marginal reticular cells and follicular dendritic cells, which may further contribute to age-related immune dysfunction. Finally, examining SLO structural features, including size, fibrosis, stiffness, and pigmentation, revealed degenerative changes that impair immune function. Collectively, this study will assist with the development of strategies aimed at delaying aging and treating age-related diseases.

## Introduction

Population aging is a worldwide trend that poses significant challenges to public health and healthcare systems. As individuals grow older, the immune system undergoes a decline known as immunosenescence [1]. This decline hampers the body’s ability to combat infections, making older populations more vulnerable to autoimmune diseases, infectious diseases, cardiovascular diseases, and cancers, etc [2–4]. Therefore, there is an urgent need to deepen our understanding of the immune system’s age-related alterations and their underlying mechanisms to facilitate development of anti-aging interventions and therapies for age-related diseases [5]. Recent studies have increasingly illuminated the age-associated changes occurring within the microenvironment of primary lymphoid organs, such as the bone marrow and the thymus [6–8]. For instance, age-related thymus degeneration reduces thymocyte populations while disrupting the thymic stromal microenvironment [9, 10]. These primary lymphoid organs, critical to the development and maturation of immune cells, deliver lymphocytes to peripheral lymphoid organs and provide the foundational framework for the immune response. Complementing this, secondary lymphoid organs (SLOs), play a pivotal part in the execution of immunological responses by mature immune cells [11]. Together, primary and secondary lymphoid organs form a complex, interconnected network essential for sustaining effective immune function. Despite the substantial progress in understanding the aging of primary lymphoid organs, studies investigating age-related changes in SLOs remain relatively limited.

SLOs function as critical filtering and surveillance systems, playing an essential role in initiating adaptive immune responses. These organs encompass the spleen, lymph nodes (LNs), Peyer’s patches (PPs), and other mucosa-associated lymphoid tissues [12]. The spleen, located in the abdomen, acts as a blood filter by capturing blood-borne pathogens. LNs are extensively dispersed throughout the body and serve as lymph filters where lymphatic vessels converge at non-mucosal sites. PPs, situated within the intestinal mucosa, serve a key role in initiating immune responses against pathogens that penetrate the intestinal mucosal barrier [12]. These organs are located throughout the body to capture pathogens that enter via different routes, whether through tissues or the bloodstream, and create environments that support the activation and multiplication of mature immune cells. Therefore, elucidating the structural and functional alterations of SLOs during the aging process is one of the core scientific issues for a deeper understanding of immunosenescence. Among these organs, the LNs and spleen have attracted the most attention, particularly in relation to age-associated changes in immune cell populations, including alterations in the proportions of immune cell subpopulations and the effects of dysregulated chemokine expression, and age-related immune cell subpopulations have been progressively identified and characterised [13–16]. These changes are associated with the efficacy of immune responses, suggesting a potential causal relationship that merits further exploration. On the other hand, age-related investigations of PPs have concentrated on senescence-associated damage to microfold (M) cells, including reduced expression of maturation-associated chemokines and diminished ability to transport luminal antigens [17]. However, current findings offer only a partial understanding of aging in SLOs [18, 19]. Continued research into age-related changes in SLOs is crucial to fully understand immune alterations during aging and identify potential therapeutic targets to restore immune function in older adults.

A key characteristic of SLOs is the highly organised spatial distribution of diverse immune cell populations, which facilitates robust, antigen-specific adaptive immune responses. The compartmentalisation is maintained by stromal cell populations, which are vital for organogenesis, structural support, immunomodulation, and homeostatic maintenance in SLOs [20, 21]. Key stromal components, including fibroblastic reticular cells (FRCs), blood endothelial cells (BECs), and lymphatic endothelial cells (LECs), provide a supportive microenvironment conducive to the development, activation, and spatial organisation of immune cells. Additionally, these stromal cells secrete cytokines and chemokines that orchestrate immune responses and maintain immune homeostasis [22]. Recent research into the population structure and functional roles of stromal cells within SLOs has demonstrated that, during the process of senescence, these cells can modulate immune cell recruitment and localisation through cytokine and chemokine release [23–25]. Further understanding how stromal cell aging affects immune function may yield significant insights into immunosenescence and inform therapeutic strategies to improve immune health in aging populations.

In this work, we conducted comprehensive examinations of age-related changes in SLOs of mice, including LNs, spleen, and PPs, using a range of techniques such as single-cell RNA sequencing (scRNA-seq) data analysis, morphological assessment, flow cytometry, and immunofluorescence staining. This investigation contributes to our understanding of the intricate association between the aging process and immune system alterations, thereby helping clarify the pathogenesis of age-associated illnesses and recognition of potential targets for anti-aging interventions.

## Results

### Altered proportions and organisation of immune cells in aged SLOs

**Fig. 1:**
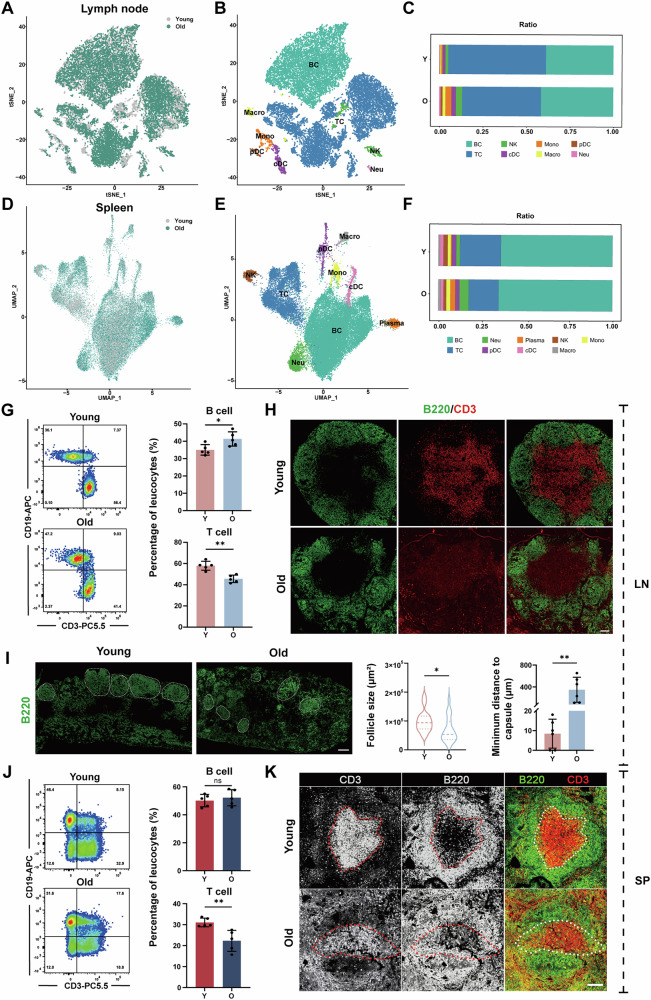
Altered proportions and organisation of immune cells in aged SLOs. **A** tSNE plot showing cell origin by colour in young and old LNs. **B** tSNE plot of cell clusters in LN tissues. **C** Chart showing the ratios of immune cell subsets in young and old LNs. **D** UMAP plot showing cell origin by colour in young and old spleen. **E** UMAP plot of cell clusters in spleen tissues. **F** Chart showing the ratios of immune cell subsets in young and old spleen. **G** Percentage of T and B cells in young and old LNs (*n* = 5 mice/group). **H** Representative immunostaining of LN for B cells (B220, green) and T cells (CD3, red). Scale bars represent 100 μm (*n* = 5 mice/group). **I** Histological sections of LNs with immunostaining for B cells (B220, green) and measurement of the area of B220^+^ B cell follicles and minimum distance to capsule in young and old mice. Scale bars represent 200 μm (*n* = 5 mice/group). **J** Percentage of T and B cells in young and old spleen (*n* = 5 mice/group). **K** Representative immunostaining of spleen for B cells (B220, green) and T cells (CD3, red). Scale bars represent 100 μm (*n* = 5 mice/group). *P*-values were calculated between two groups using an unpaired *t*-test. ns, not significant; *P < 0.05; **P < 0.01; ***P < 0.001. BC B cell, TC T cell, NK natural killer cell, cDC classical dendritic cell, Mono monocyte, Macro macrophage, pDC plasmacytoid dendritic cell, Neu neutrophil.

Senescent immune cells have been identified as among the most detrimental cell types in the aging process, accelerating the senescence of solid organs and contributing to systemic aging mechanisms [26]. To elucidate the intricate age-related changes in SLOs and the relationship between aging and the immune system, we analysed scRNA-seq data from LNs (GSA accession number CRA004687) and spleen (GSE132042) of both young and old mice. The main immune cell populations in the LNs and spleen were identified by clustering of these data (Fig. 1A, D). Based on marker gene expression (Fig. S1A, B), we pinpointed several distinct cell clusters, including B cells, T cells, and natural killer (NK) cells (Fig. 1B, E). The comparative analysis highlighted age-associated variations in immune cell populations: notably, the B cell population increased during aging, whereas the proportion of T cells decreased (Fig. 1C, F), which may elevate the risk of chronic inflammation and limit immune surveillance and response function [27, 28].

To further investigate the changes linked to the senescence of T and B cells, we collected LN and spleen samples from young and aged mice, and quantified these populations using flow cytometry. As anticipated, aged mice exhibited a relative drop in T cell numbers alongside a relative rise in B cell populations (Fig. 1G, J). Additionally, we examined potential morphological alterations using immunofluorescence staining. The results revealed that aging diminished the distinct boundaries between T and B cell regions (Fig. 1H, K), which may disrupt the signalling between these cell types and consequently diminish the efficacy of the immune response [29]. Notably, the architecture of lymphoid follicles was compromised in aged mice. Lymphoid follicles are critical for essential immune processes such as antigen presentation and germinal center (GC) reactions [30]. To investigate the implications of aging on these structures, we compared the size of the follicles and their shortest distance proximity to the capsule. Senescent follicles were found to be smaller, exhibit irregular shapes, and shift towards the medulla, a phenomenon that was less pronounced in the spleen (Fig.1I, S1C). These changes suggest that processes such as B cell maturation and antibody production may be hindered in aged individuals. In summary, our findings indicated that both the composition and organisation of immune cells within the SLOs of aged mice were significantly altered, collectively reflecting the overall decline of the immune system associated with aging.

### Altered immune cell subpopulation ratios and functions in aged SLOs

**Fig. 2:**
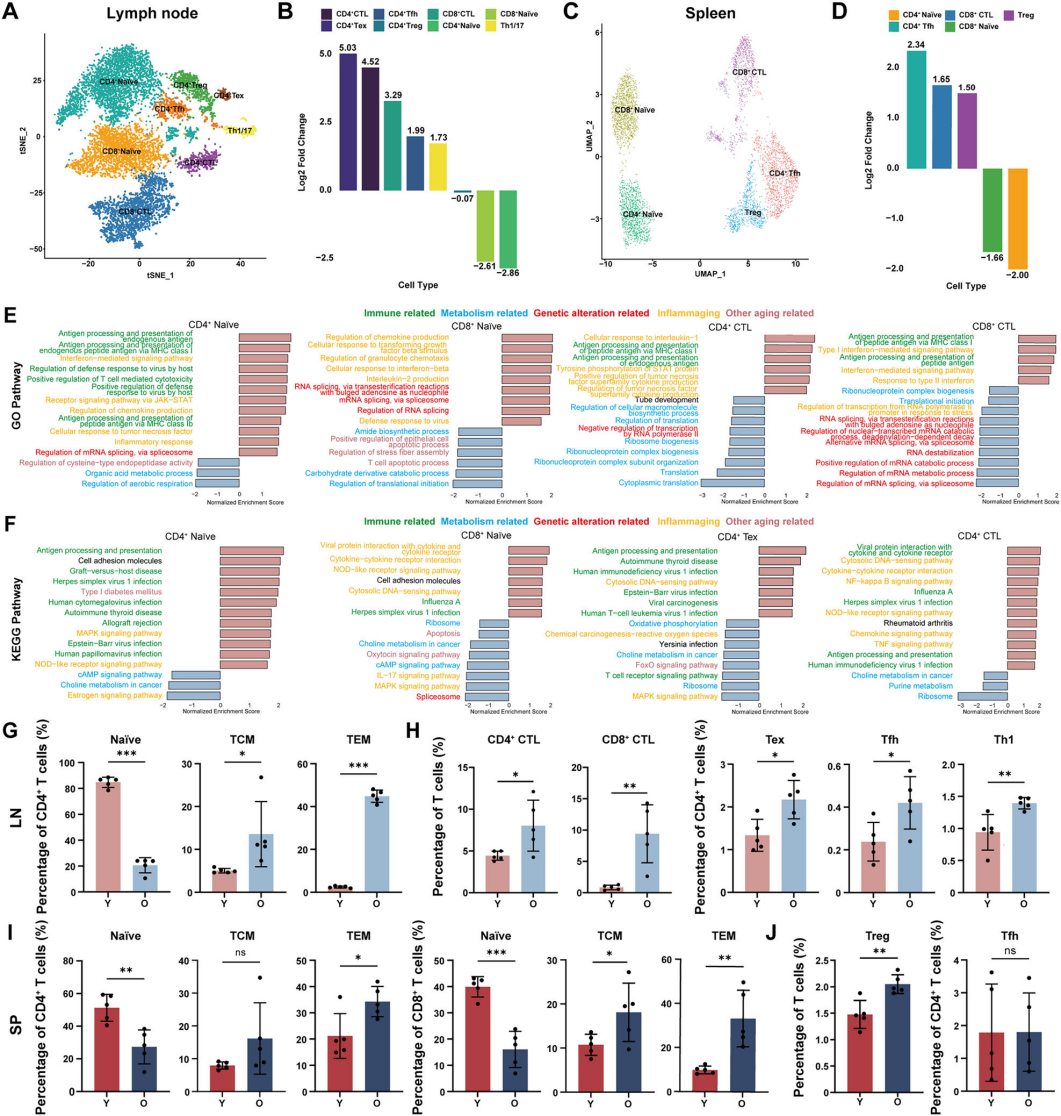
Altered immune cell subpopulation ratios and functions in aged SLOs. **A** tSNE plot showing subclusters of T cell subsets in young and old LNs. **B** Chart showing the cell ratios of subclusters of T cell subsets in young and old LNs. **C** UMAP plot showing subclusters of T cell subsets in young and old spleen. **D** Chart showing the cell ratios of subclusters of T cell subsets in young and old spleen. **E**, **F** Normalised Enrichment Score (NES) of enrichment for GO biological process terms and KEGG terms with an age-related change. Only top 15 GO or KEGG terms with *P*-value < 0.05 were listed for major T cell subsets. **G** Flow cytometric analysis of naïve CD4^+^ T cells (CD45^+^, CD3^+^, CD4^+^, CD62L^+^, CD44^-^), TCM cells (CD45^+^, CD3^+^, CD4^+^, CD62L^+^, CD44^+^), TEM cells (CD45^+^, CD3^+^, CD4^+^, CD62L^-^, CD44^+^) was performed. The percentage representation of the populations was shown in young and old LNs (*n* = 5 mice/group). **H** Flow cytometric analysis of CD4^+^ CTLs (CD4^+^, GZMB^+^), CD8^+^ CTLs (CD8a ^+^, GZMB^+^), Tex cells (CD45^+^, CD3^+^, CD4^+^, PD-1^+^, TIM3^+^), Tfh cells (CD45^+^, CD3^+^, CD4^+^, CD154^+^, CXCR5^+^) and Th1 cells (CD45^+^, CD3^+^, CD4^+^, CXCR3^+^) was performed. The percentage of the populations was shown in young and old LNs (*n* = 5 mice/group). **I** Flow cytometric analysis of naïve CD4^+^ T cells, TCM cells, TEM cells, naïve CD8^+^ T cells (CD45^+^, CD3^+^, CD8a^+^, CD62L^+^, CD44^-^), TCM cells and TEM cells was performed. The percentage of the populations was shown in young and old spleen (*n* = 5 mice/group). **J** Flow cytometric analysis of Treg cells (CD4^+^, FOXP3^+^) and Tfh cells was performed. The percentage of the populations was shown in young and old spleen (*n* = 5 mice/group). *P*-values were calculated between two groups using an unpaired *t*-test. ns, not significant; *P < 0.05; **P < 0.01; ***P < 0.001.

Aging has been demonstrated to affect the immune system by modifying the distribution of immune cell subpopulations [31]. In our study, we investigated the changes in T cell subsets in elderly mice, and found a large reduction in the percentages of CD4⁺ and CD8⁺ naïve T cells within the LNs and spleen, whereas populations such as cytotoxic T lymphocytes (CTLs) and regulatory T (Treg) cells exhibited varying degrees of increase (Fig. 2A–D). Subsequently, we analysed the senescence-associated differentially expressed genes (DEGs) across T and B cell subsets. Through Gene Ontology (GO) and Kyoto Encyclopedia of Genes and Genomes (KEGG) enrichment analyses, we observed that the DEGs were predominantly enriched in metabolic, immune and other aging-related pathways [32–34] (Fig. 2E, F, S2A, B, G, H), suggesting a strong association between T cell subsets and the aging process. Similarly, our assessment of gene expression profiles in B cell populations under senescent conditions indicated a strong correlation between B cell subsets and aging (Fig. S2C, I). Collectively, these findings indicated that T cells were particularly susceptible to the aging process, resulting in diminished immune functionality with advancing age.

Previous studies have indicated that T cells undergo age-related changes that progressively impair their physiological functions [28]. To delve deeper into this phenomenon, we first examined the effects of aging on T cell subpopulations within the LNs and spleen using flow cytometry. CD4^+^ T cells are crucial for orchestrating adaptive immune responses [32, 35], whereas CD8^+^ T cells play a pivotal part in immune defence by identifying and destroying virus-infected or cancerous cells. Our results showed that CD4^+^:CD8^+^ ratio decreased during aging (Fig. S2D, J), which may be attributable to reduced thymic production of new T cells and T cell malfunction. As suggested by the results of the cell ratio analysis, we detected the senescence-related alterations in these T cell subpopulations. We observed the proportions of naïve CD4^+^ and CD8^+^ T cells decreased significantly in old LNs (Fig. 2G, S2E), suggesting diminished naïve T cell production as a consequence of thymic degeneration. Then, we further focused on alterations in CD4^+^ cell subsets that occur with aging. The results showed an increase in the proportion of CD4^+^ and CD8^+^ CTLs, CD4^+^ exhausted T (Tex) cells, CD4^+^ follicular helper T (Tfh) cells, and CD4^+^ T helper 1 (Th1) cells in LNs following senescence (Fig. 2H). Similarly, we assessed the proportions of T cell subpopulations in the spleen and noted a significant increase in the Treg, Tex and Th1 subsets (Fig. 2I, J, S2K). The alterations in these T cell subpopulations, which are crucial for maintaining immune homeostasis and preventing disease, indicated that the organism was experiencing a state of chronic inflammation or sustained antigenic stimulation [36, 37].

In addition, we examined alterations in other important subpopulations of T cells using flow cytometry. The results indicated an increase in the proportion of central memory T (TCM) cells and effector memory T (TEM) cells (Fig. 2G, I, S2E), suggesting that aging may result in chronic exposure to various pathogens or persistent infections, thereby contributing to the accumulation of memory cells. Previous research reported significant defects in the B cell compartment with aging [38]. Consistent with these findings, we observed that during aging, the proportion of naïve B cells decreased and the number of memory B cells expanded (Fig. S2F, L), indicating a narrowing of the naïve B cell pool and/or an accumulation of immune memory. More specifically, there was a decrease in unswitched B cells alongside an increase in switched B cells, suggesting that aged individuals may have encountered more infections, leading to enhanced immune memory and a more mature immune response capable of producing antibodies with higher affinity. Overall, these results demonstrated that aged SLOs exhibited altered ratios and functions of immune cell subpopulations, indicating a disrupted immune status and reduced immune functionality in the organism.

### Accumulation of senescent immune cells in aged SLOs

**Fig. 3:**
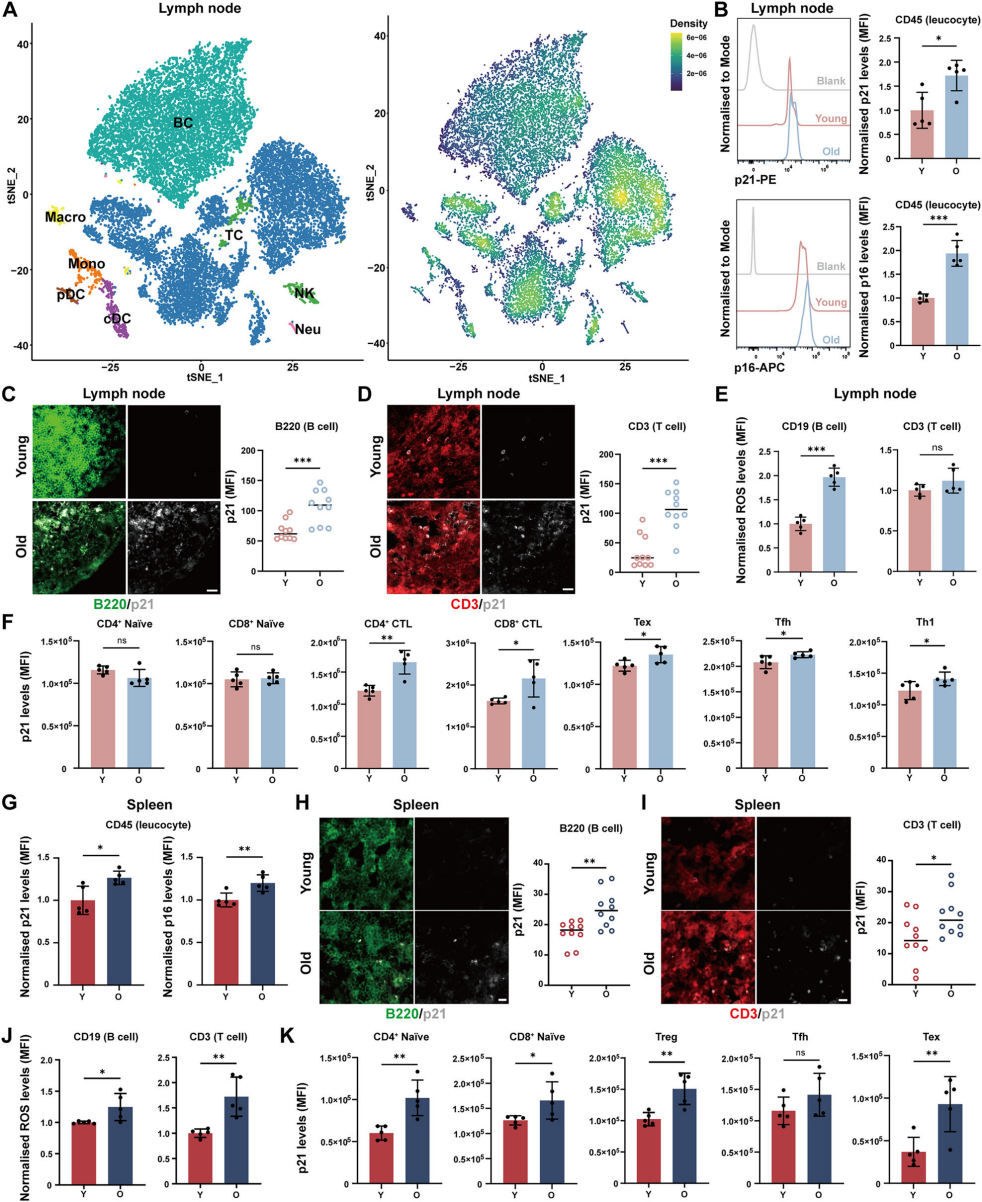
Accumulation of senescent immune cells in aged SLOs. **A** *Left Panel:* tSNE plot illustrating subclusters of cell subsets in young and old LNs; *Right Panel:* Density distribution plot of aging scores. **B** Flow cytometric analysis of p21 and p16 levels in CD45^+^ leucocyte populations in young and old LNs (*n* = 5 mice/group). **C** Histological sections of LNs with immunostaining for B cells (B220, green) and measurement of p21 MFI of B220^+^ B cell area in young and old LNs. Scale bars represent 20 μm (*n* = 5 mice/group). **D** Histological sections of LNs with immunostaining for T cells (CD3, red) and measurement of p21 MFI of CD3^+^ T cell area in young and old LNs. Scale bars represent 20 μm (*n* = 5 mice/group). **E** Flow cytometric analysis of ROS levels in CD19^+^ B cell and CD3^+^ T cell populations in young and old LNs (*n* = 5 mice/group). **F** Flow cytometric analysis of p21 levels in naïve CD4^+^ T cells, naïve CD8^+^ T cells, CD4^+^ CTLs, CD8^+^ CTLs, Tex cells, Tfh cells and Th1 cells populations in young and old LNs (*n* = 5 mice/group). **G** Flow cytometric analysis of p21 and p16 levels in CD45^+^ leucocyte populations in young and old spleen (*n* = 5 mice/group). **H** Histological sections of spleen with immunostaining for B cells (B220, green) and measurement of p21 MFI of B220^+^ B cell area in young and old spleen. Scale bars represent 10 μm (*n* = 5 mice/group). **I** Histological sections of spleen with immunostaining for T cells (CD3, red) and measurement of p21 MFI of CD3^+^ T cell area. Scale bars represent 10 μm (*n* = 5 mice/group). **J** Flow cytometric analysis of ROS levels in B cell and T cell populations in young and old spleen (*n* = 5 mice/group). **K** Flow cytometric analysis of p21 levels in naïve CD4^+^ T cells, naïve CD8^+^ T cells, Treg cells, Tfh cells and Tex cells in young and old spleen (*n* = 5 mice/group). *P*-values were calculated between two groups using an unpaired *t*-test. ns not significant, *P < 0.05; **P < 0.01; ***P < 0.001. BC B cell, TC T cell, NK natural killer cell, cDC classical dendritic cell, Mono monocyte, Macro macrophage, pDC plasmacytoid dendritic cell, Neu neutrophil.

Senescence affects immune cell differentiation and function [39], thereby diminishing their capacity to combat viral and bacterial pathogens and hindering their ability to efficiently repair cellular damage. To assess the extent of senescence in major immune cell populations, we first performed senescence scoring on LNs and spleen. The results revealed a pronounced manifestation of senescence within the T and B cell populations (Fig. 3A, S3A). To further elucidate the effects of aging on various immune cell subgroups in SLOs, we analysed the expression of senescence markers [40] using flow cytometry. We noted an elevation in the levels of both p16^INK4a^ and p21^Waf1/Cip1^ in leucocytes (CD45^+^) (Fig. 3B). Meanwhile, we employed immunofluorescence to measure the expression of p21^Waf1/Cip1^ in immune cell populations. We found that the p21^Waf1/Cip1^ signal was higher in aged mice, partially coinciding with B220 and CD3 markers. Additionally, the mean fluorescence intensity (MFI) of p21^Waf1/Cip1^ was significantly elevated in both T and B cells with aging (Fig. 3C, D). Previous studies have identified increased reactive oxygen species (ROS) production as a hallmark of senescent cells [41]. In light of this, we examined the ROS levels in immune cell populations via flow cytometry and observed an increase in the MFI of ROS in both T and B cells (Fig. 3E). Moreover, we assessed the expression levels of p21^Waf1/Cip1^ in key T cell subpopulations, and the results showed increased senescence marker expression in most populations, although this was not significant in the naïve T cell population (Fig. 3F). Based on these observations, we proposed that T cells and B cells in the LNs of aged mice undergo significant senescence. Similarly, we investigated the extent of senescence of the immune cells in the spleen. The results indicated that the MFI of p16^INK4a^ and p21^Waf1/Cip1^ were increased in leucocytes (Fig. 3G), with significant elevations in p21^Waf1/Cip1^ levels in T and B cells with advancing aging (Fig. 3H, I). Meanwhile, the MFI of ROS in both B and T cell populations was found to be elevated (Fig. 3J). The expression of p21^Waf1/Cip1^ in those important T cell subpopulations within the spleen also showed an increase (Fig. 3K, S3B). Taken together, these results suggested that aging facilitated the accumulation of senescent immune cells in SLOs. The rise in immunosuppressive cells, the decline in cytotoxic cell functionality, and the upregulation of immune checkpoint molecules during aging contribute to impaired immune surveillance, which could further exacerbate the accumulation of senescent cells, thereby creating a vicious loop that intensifies the decline in immune function associated with senescence [42–44].

### Altered distribution of MRC and diminished FDC networks in aged SLOs

Stromal cells within SLOs play a pivotal part in organ development, providing structural support, regulating immune functions, maintaining compartmentalisation, and ensuring homeostasis [45, 46]. FRCs, which are specialised fibroblasts of mesenchymal origin, include subsets such as follicular dendritic cells (FDCs) that support B cell follicles and marginal reticular cells (MRCs) that primarily support macrophages. Given the important role of stromal cells in organizing structure and mediating immune responses in SLOs, we proposed that age-associated changes in these stromal cells may be linked to the immunosenescence observed in SLOs. To investigate this, we first quantified the expression levels of p16^INK4a^ and p21^Waf1/Cip1^ in LN stromal cells via flow cytometry, revealing elevated levels of both markers in stromal cells (Fig. S4A). Meanwhile, we examined the extent of senescence in the stromal cells through immunofluorescence, which indicated an increased signal for p21^Waf1/Cip1^ in the aged mice, partially coinciding with ERTR7, a fibroblast marker (Fig. S4B). The ROS levels were also evaluated, and the results revealed that the MFI of ROS in FRCs and LECs was increased (Fig. S4C). Together, these findings suggested significant aging of stromal cells in the LNs of aged mice.

**Fig. 4:**
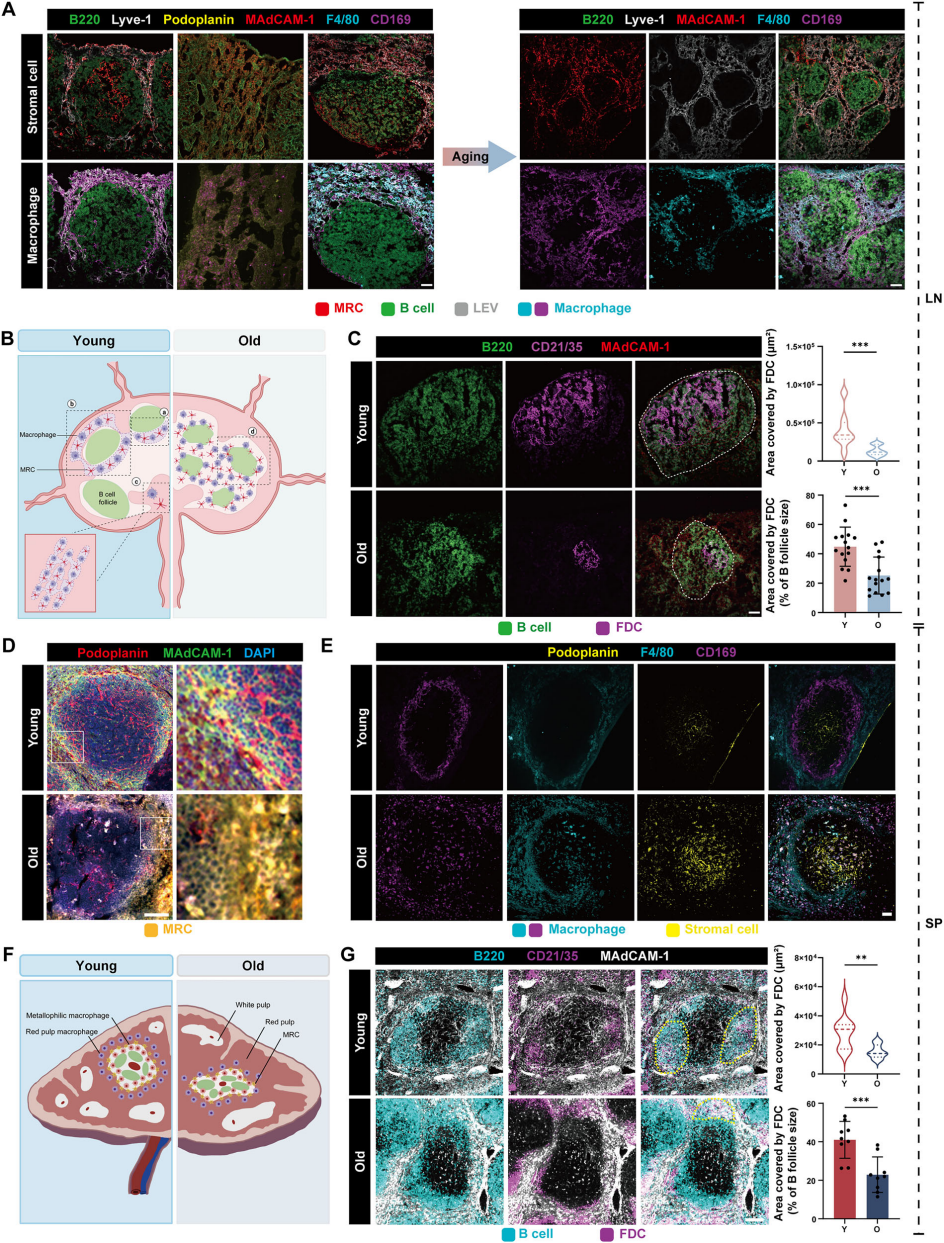
Altered distribution of MRC and diminished FDC networks in aged SLOs. **A** Histological sections of LNs tissues with immunostaining for MRCs (MAdCAM-1, red), B cells (B220, green), LEV cells (Lyve-1, gray) and macrophages (F4/80, strong cyan; CD169, magenta) in young and old mice. Scale bars represent 50 μm. **B** Schematic diagram of the distribution of MRCs and macrophages in young and old LNs (created with BioRender.com). **C** Histological sections of LNs tissues with immunostaining for FDCs (CD21/35, magenta) and B cells (B220, green), and measurement of FDC area in young and old mice. Scale bars represent 50 μm (*n* = 5 mice/group). **D** Histological sections of spleen with immunostaining for MRCs (MAdCAM-1, green; podoplanin, red) in young and old mice. Scale bars represent 100 μm. **E** Histological sections of spleen with immunostaining for macrophages (F4/80, strong cyan; CD169, magenta) and stromal cells (podoplanin, yellow) in young and old mice. Scale bars represent 50 μm. **F** Schematic diagram of the distribution of MRCs and macrophages in young and old spleen (created with BioRender.com). **G** Histological sections of spleen with immunostaining for FDCs (CD21/35, magenta) and B cells (B220, strong cyan) and measurement of FDC area in young and old mice. Scale bars represent 100 μm (*n* = 5 mice/group). *P*-values were calculated between two groups using an unpaired *t*-test. ns not significant; *P < 0.05; **P < 0.01; ***P < 0.001.

Further, we assessed the age-related alterations in the distribution of stromal cells within the LNs through immunofluorescence. The results demonstrated the altered distribution of MRCs location with aging, changed from three distinct structures observed in younger individuals, including the cord structure in the medullary sinus area, the enveloping-like structure and the cup structure in the subcapsular sinus area, to a more scattered distribution between the follicles in older individuals (Fig. 4A,B). The results also revealed that the alteration of macrophage distribution during aging was highly consistent with MRCs. In young LNs, macrophages are typically distributed in the subcapsular sinus, marginal sinus, and medulla [47]. However, with aging, we observed a shift in their distribution towards the interfollicular space, suggesting a gradual loss of the medullary sinus architecture in aging LNs, accompanied by a corresponding relocation of functional regions. Furthermore, an obvious reduction in the proportion of FDCs within follicles was noted (Fig. 4C), which may adversely affect GC formation and be related to the decreased quality and efficiency of the immune response [48].

In a similar vein, we found evidence of stromal cell aging in the spleen of elderly mice (Fig. S4D–F). Immunofluorescence analysis revealed that the marginal zone region of the aged spleen exhibited increased thickness and discontinuity, with loosely distributed MRCs that failed to maintain a continuous boundary along the marginal zone (Fig. 4D). Marginal zone macrophages are predominantly distributed in the white pulp and red pulp areas [49]. Similarly, we observed significant structural disorganisation of marginal zone macrophages associated with aging (Fig. 4E, F). Specifically, the distribution of macrophages within the inner layer of the marginal zone was disrupted in older spleen, with macrophages no longer localised adjacent to the marginal zone but rather dispersed throughout, with some even spilling over into neighbouring regions. Additionally, the distribution of red pulp macrophages exhibited a similar degree of disarray (Fig. 4E, F), indicating the potential existence of macrophage-stromal cell circuits within the spleen. Furthermore, we noted a reduction in the area occupied by FDCs, with cells within the follicles becoming increasingly dispersed (Fig. 4G). These structural changes could hinder the capacity of B cells to mount effective responses to antigens, subsequently impacting both antibody production and the maintenance of immune memory. Overall, our findings indicated that aging was associated with altered distribution patterns of MRCs, changes in macrophage localisation, and a diminished FDC network within SLOs. Such alterations may affect the capture of immune complexes, impede GC formation, disrupt antigen presentation, and ultimately contribute to a decline in immune responsiveness [50, 51].

### The impact of aging on morphology, fibrosis and stiffness of SLOs

**Fig. 5:**
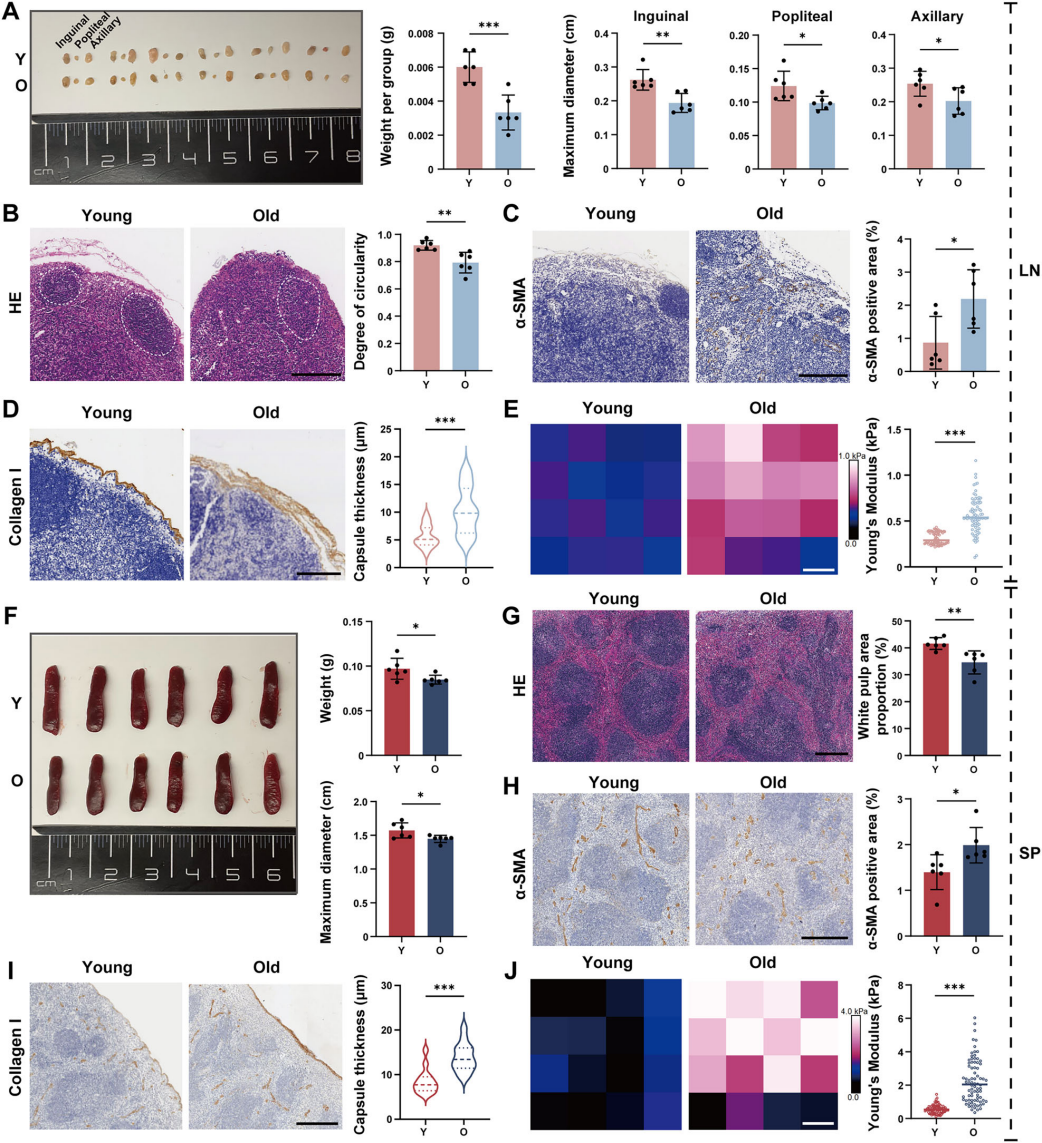
The impact of aging on morphology, fibrosis and stiffness of SLOs. **A** Representative images of inguinal, popliteal and axillary LNs from young and old mice and measurement of weight of LNs in each group. Each group comprised one LN from each of the specified anatomical locations, and measurement of the maximum diameter of each LN (*n* = 6 mice/group). **B** Representative images of LN tissue sections after HE staining and measurement of degree of circularity of LNs in young and old mice. Scale bars represent 200 μm (*n* = 6 mice/group). **C** Representative IHC images of LN tissues and quantitative analysis of α-SMA positive area. Scale bars represent 200 μm (*n* = 6 mice/group). **D** Representative IHC images of LN tissues and quantitative analysis of the thickness of capsule (collagen I^+^ area). Scale bars represent 200 μm (*n* = 6 mice/group). **E** Atomic force images and statistical analysis in LN tissues. Scale bars represent 1.0 μm (*n* = 5 mice/group). **F** Representative images of spleen from young and old mice and measurement of weight and maximum diameter of each spleen (*n* = 6 mice/group). **G** Representative images of white pulp sections after HE staining and measurement of white pulp area in young and old mice. Scale bars represent 300 μm (*n* = 6 mice/group). **H** Representative IHC images of spleen tissues and quantitative analysis of α-SMA positive area. Scale bars represent 300 μm (*n* = 6 mice/group). **I** Representative IHC images of spleen tissues and quantitative analysis of the thickness of capsule (collagen I^+^ area). Scale bars represent 300 μm (*n* = 6 mice/group). **J** Atomic force images and statistical analysis in spleen tissues. Scale bars represent 1.0 μm (*n* = 5 mice/group). *P*-values were calculated between two groups using an unpaired *t*-test. ns not significant; *P < 0.05; **P < 0.01; ***P < 0.001.

Senescent cells exhibit altered metabolic profiles, cell cycle arrest, and senescence-associated secretory phenotype (SASP) activation, all of which can influence tissue regeneration, repair, and homeostasis [52]. These characteristics are often associated with age-related alterations in various tissues. To further investigate the morphological and structural changes of SLOs during aging, we compared LNs and spleen from young and old mice. Our findings revealed that aging was associated with a reduction in LN weight and a decrease in the maximum diameters of the inguinal, popliteal, and axillary LNs (Fig. 5A). Histopathological analysis of lymphoid tissues demonstrated a decrease in follicle circularity, with a more dispersed morphology (Fig. 5B), likely attributable to a reduction in lymphocyte populations and degradation of the microstructural integrity. In addition, we observed increased pigmentation in the LNs with aging, especially in the subcapsular sinus and medullary sinus regions (Fig. S5A). We hypothesised that this phenomenon may be linked to the accumulation of lipofuscin, the pigment commonly associated with cellular damage and oxidative stress. The presence of this pigment in senescent LNs may reflect the byproducts of prolonged immune cell activity and tissue repair processes. Furthermore, we measured the expression levels of α-smooth muscle actin (α-SMA) and collagen I. The findings showed that α-SMA and collagen I levels increased (Fig. 5C, D), suggesting that the LNs of old mice undergo changes of capsule thickening and increased fibrosis. To assess the changes in the stiffness of LNs, we compared the elastic modulus between young and old LNs, finding a significant increase in the elastic modulus with aging (Fig. 5E). This increase likely reflected the augmented fibrosis, altered cellular composition, and changes in the metabolism and structural composition of the extracellular matrix in aging LNs.

Similarly, we noted a reduction in both the weight and the maximum diameter of the spleen with advancing age (Fig. 5F). Histopathological examination of the spleen revealed that young mice possessed well-developed white pulp, round or oval splenic bodies, periarterial lymphatic sheaths adjacent to the splenic bodies, and clear boundaries between white and red pulp. In contrast, older mice displayed a reduction in white pulp and structural disorganisation, with the white pulp primarily dispersed in strips or small clusters (Fig. 5G). Additionally, we found distinct pigmentation in the spleen associated with aging, particularly in the subcapsular sinus region and red pulp regions (Fig. S5B). We speculated that this pigmentation may be due to the accumulation of hemosiderin or lipofuscin, potentially linked to metabolic dysregulation and chronic inflammation within the spleen with aging. We also examined the levels of fibrosis and stiffness alterations in the spleen, finding elevated levels of collagen I and α-SMA (Fig. 5H, I), along with a significant increase in the elastic modulus in aged spleen tissue (Fig. 5J). Together, these findings showed that aging in SLOs was linked to increased fibrosis, enhanced stiffness, and impaired immune function, with tissue-specific morphological changes reflecting variations in immune status and function.

### Age-related changes in Peyer’s patches

**Fig. 6:**
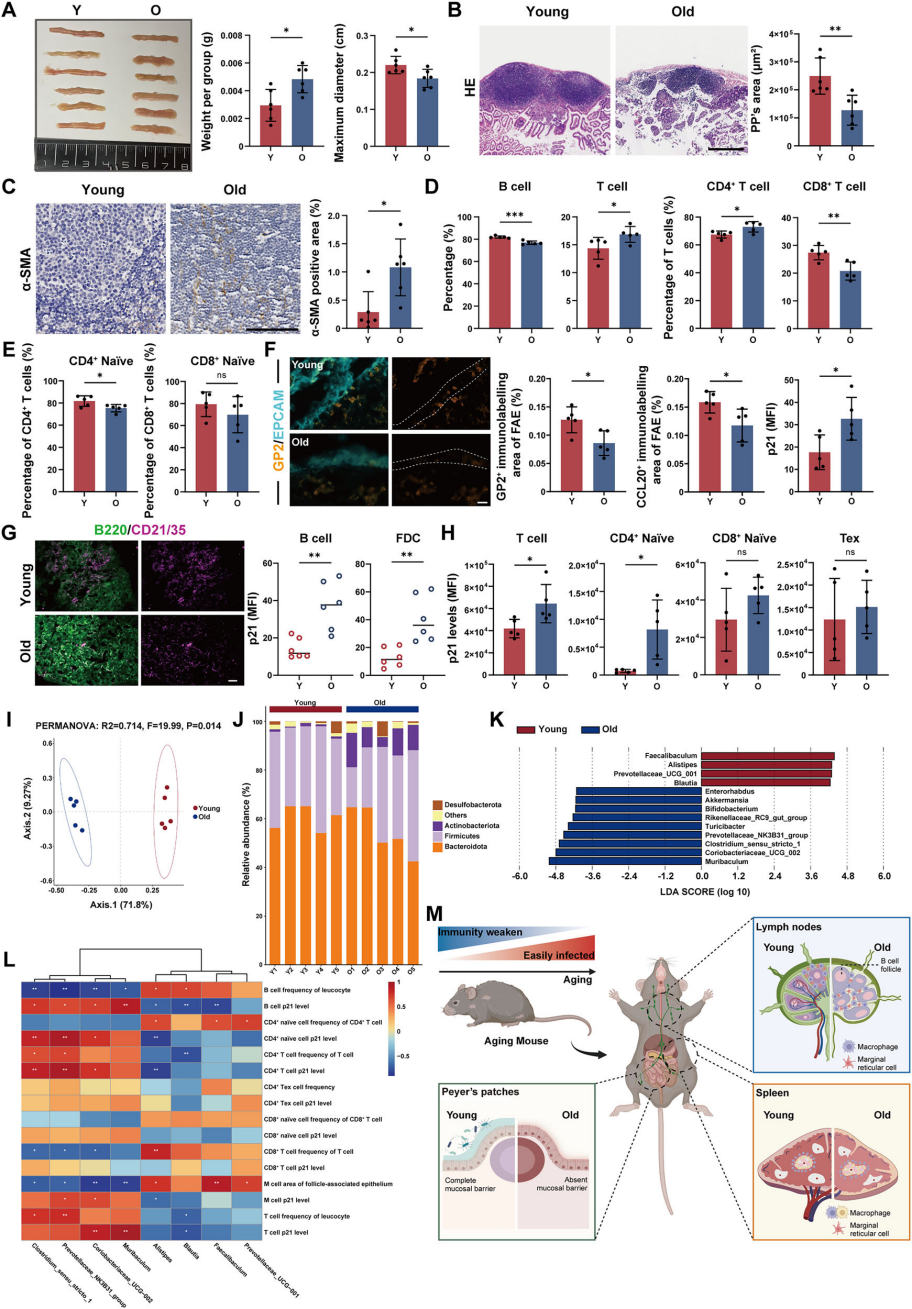
Age-related changes in Peyer’s patches. **A** Representative images of intestinal PPs and measurement of weight and maximum diameter of each intestinal PP from young and old mice (*n* = 6 mice/group). **B** Representative images of PP tissue after HE-staining and measurement of PP area in young and old mice. Scale bars represent 400 μm (*n* = 6 mice/group). **C** Representative IHC images of intestinal PPs and quantitative analysis of α-SMA positive area. Scale bars represent 100 μm (*n* = 6 mice/group). **D** Flow cytometric analysis of B cells, T cells, CD4^+^ T cells and CD8^+^ T cells was performed. The percentage representation of the populations was shown in young and old intestinal PPs (*n* = 5 mice/group). **E** Flow cytometric analysis of naïve CD4^+^ T cells and naïve CD8^+^ T cells was performed. The percentage representation of the populations was shown in young and old intestinal PPs (*n* = 5 mice/group). **F** Histological sections of intestinal PPs with immunostaining for mature M cells (GP2, bright orange) and epithelial cells (EPCAM, soft cyan), and measurement of mature M cell area in FAE and p21 MFI of mature M cell area in young and old intestinal PPs. Scale bars represent 20 μm (*n* = 5 mice/group). **G** Histological sections of intestinal PPs with immunostaining for FDCs (CD21/35, magenta) and B cells (B220, green), and measurement of p21 MFI of FDC and B cell area in young and old intestinal PPs. Scale bars represent 50 μm (*n* = 5 mice/group). **H** Flow cytometric analysis of p21 levels in T cells, naïve CD4^+^ T cells, naïve CD8^+^ T cells and Tex cells was performed. The percentage representation of the populations was shown in young and old intestinal PPs (*n* = 5 mice/group). **I** Principal coordinate analysis (PCoA) plot showing the gut microbiota composition in both young and old mice (*n* = 5 mice/group). **J** Relative abundance of bacterial phyla in young and old mice (*n* = 5 mice/group). **K** Linear discriminant analysis effect size (LEfSe) analysis identifying differentially abundant bacterial genera between young and old mice (*n* = 5 mice/group). **L** Heatmap showing the correlations between differentially abundant gut bacterial genera and cellular subpopulations in the intestinal PPs (*n* = 5 mice/group). **M** Schematic illustration of changes in LNs, spleen and intestinal PPs between young and old mice (created with BioRender.com). *P*-values were calculated between two groups using an unpaired *t*-test. ns not significant; *P < 0.05; **P < 0.01; ***P < 0.001.

The gut-associated lymphoid tissue (GALT), a major component of the intestinal mucosal immunological barrier system, is vital for the body’s defence against pathogen invasion [53]. Intestinal PPs, an important part of GALT, are considered as primary sites for the induction of mucosal immune responses. To explore the age-related changes, we collected PPs from young and aged mice. The findings indicated that with aging, the weight of PPs increased and their maximal diameter decreased (Fig. 6A). This phenomenon may be attributed to heightened tissue density due to alterations in the extracellular matrix, intestinal atrophy, and lumen width with advancing age. Histopathological analysis of PPs revealed that young mice exhibited more distinct lymphoid GCs, whereas aged mice displayed a significant reduction in the area of PPs and less defined GCs (Fig. 6B), suggesting a decline in immune function. Additionally, we observed increased levels of α-SMA (Fig. 6C), indicative of heightened fibrosis. To further investigate whether the behaviour of immune cells was consistent across all SLOs, we examined the ratios of T and B cells in PPs by flow cytometry. The results showed that with aging, the proportion of B cells decreased while the percentage of T cells increased. Furthermore, the proportion of CD8^+^ T cells decreased, while the percentage of CD4^+^ T cells increased (Fig. 6D), indicating that the distribution of immune cell subpopulations during aging was tissue-specific. Similar to our findings in LNs and spleen, we examined several important T cell subsets in PPs (Fig. 6E, S6A). We found a decrease in the proportion of naïve CD4^+^ T cells with age, while the decrease in naïve CD8^+^ T cells was not statistically significant. M cells, located in follicle-associated epithelium (FAE), facilitate antigen transcytosis and transport antigens to PPs, which is important for inducing effective immune responses to mucosal antigens [54–56]. To investigate age-related alterations in M cells, we assessed the expression of glycoprotein 2 (GP2) and CCL20, which are markers of mature M cells [57–60], through immunofluorescence staining. The results showed that the area occupied by mature M cells in the FAE was significantly reduced in aged mice compared to young mice (Fig. 6F, S6B), suggesting that M cells underwent significant senescence with aging. We also observed less FDC distribution within the B follicles of senescent PPs (Fig. 6G). To detect the senescence of these cell populations in PPs, we measured the expression levels of p21^Waf1/Cip1^ in immune cells and stromal cells by flow cytometry and immunofluorescence staining. The results demonstrated that these populations exhibited elevated levels of p21^Waf1/Cip^, signifying an accumulation of senescence in the aged PPs (Fig. 6F–H).

We speculated that the observed changes may be attributed to the direct interaction of PPs with the intestinal microbiota. Previous studies suggested that intestinal microbiota can affect intestinal GC B cells and Treg cells, playing a pivotal part in the regulation of intestinal T cell homeostasis [61–64]. Additionally, it has been shown that gut microbiota may impact the formation of M cells [65]. In this study, we collected faecal samples from both young and aged mice and employed 16S rRNA sequencing to delve into their gut microbiota composition. The two groups’ microbiota composition differed significantly, according to principal coordinate analysis (PCoA) (Fig. 6I). At the phylum level, aged mice exhibited a higher abundance of Actinobacteriota and a lower Firmicutes to Bacteroidetes (F/B) ratio (Fig. 6J). At the genus level, aged mice had an increased relative abundance of *Muribaculum*, *Coriobacteriaceae*_UCG_002, and *Clostridium*_sensu_stricto_1, whereas young mice exhibited a higher prevalence of *Faecalibaculum*, *Alistipes*, and *Prevotellaceae*_UCG_001 (Fig. 6K). To investigate the association between gut microbiota and B cells, T cells, and M cells in the aging organism, we performed Spearman correlation analysis to identify the patterns of correlation between these factors (Fig. 6L). Certain indicators of PP cell types, such as the p21^Waf1/Cip1^ levels of B cells and the M cell area of the FAE, were found to be correlated with the abundance of specific bacterial genera in the gut microbiota. Several studies have reported that faecal microbiota transplantation (FMT) experiments can regulate the intestinal microbiota of recipients, and have been employed to investigate the role of microbiota in improving spermatogenic function decline in elderly mice [66, 67]. Through our FMT experiment (Fig. S6C), we observed that the gut microbiota from old donors affected the aging process of the recipients’ PPs. Specifically, recipient mice that received microbiota from old donors (old-FMT recipients) exhibited elevated levels of ROS in immune cells and increased expression of p21^Waf1/Cip1^ in tissue sections compared to recipient mice that received microbiota from young donors (young-FMT recipients) (Fig. S6D, E). These results established preliminary causal evidence supporting the role of the gut microbiota-PPs axis in age-related immunosenescence. In summary, these findings indicated that senescence was associated with alterations in the morphology, fibrosis, and immune function of PPs, as well as the accumulation of senescent cells, with these age-related changes potentially being influenced by the gut microbiota.

## Discussion

Aging is a progressive, time-dependent physiological process that results in a decline in biological function and an increased vulnerability to a variety of diseases. In the aging process, the immune system is often characterised by chronic inflammation and a diminished defensive capacity, which may contribute to the pathogenesis of various age-associated illnesses [68, 69]. Immunosenescence is emerging as a potential key breakthrough in mitigating or reversing aging-related degeneration, with numerous exogenous and endogenous “anti-aging” molecules currently under investigation for their ability to extend healthy lifespan [70]. As integral components of the immune system, SLOs serve as a crucial role in the interaction and activation of immune cells. Exploring age-related changes in SLOs is vital for acquiring a deeper understanding of immune system alterations during aging and serves as a key research area for developing anti-aging therapies [71]. In this study, we conducted a systematic examination of age-related changes in LNs, spleen, and PPs, encompassing morphological changes in organ architecture, spatial reorganisation of cellular populations, quantitative assessments of senescence marker expression, and precise identification of key cell subsets. Through an integrative analysis of multidimensional datasets, this work provides a more comprehensive experimental foundation for elucidating the functional dynamics and complexity of the immune system during the aging process. Furthermore, it enhances our understanding of immunosenescence and informs the development of immunotherapeutic strategies for age-associated diseases.

The characteristics of aging have been extensively studied, such as telomere shortening, chronic inflammation, stem cell exhaustion, altered cellular communication, and epigenetic alterations [72]. Our investigation revealed that alterations in immune cell types within SLOs are evident during the aging process and may contribute to the associated decline in immunological function. Previous studies have predominantly focused on aging-related alterations in primary lymphoid organs, including the myeloid differentiation preference of hematopoietic stem cells in the bone marrow and the diminished production of naïve T cells by the thymus [73, 74]. A recent study identified SOX4^+^ naïve T cell populations as potential markers of thymic senescence within the human immune system [75]. In terms of alterations occurring in SLOs, most research has focused on assessing the ratios of B cells to T cells, the proportions of CD4^+^ and CD8^+^ T cells, and the balance between memory and naïve T cells [23, 24, 76]. In contrast, our study not only assessed these commonly examined immune cell subpopulations but also investigated alterations in the ratios of T cell subsets, including Treg and Tfh cells, based on our scRNA-seq data analysis. We observed that T cell subpopulations underwent reorganisation with age, exhibiting distinct functional characteristics that may serve as indicators of declining immune function. Specifically, we noted a decline in naïve T cells within LNs, spleen, and PPs, accompanied by increases in populations of Tex, Tfh, Th1, Treg, and CTL. Concurrently, gene expression analysis indicated that these subsets are enriched in metabolic, immune and other aging-related pathways, thereby linking them to immunosenescence. To further investigate the senescence characteristics of immune cell populations, we measured the expression levels of several key senescence markers and confirmed the accumulation of senescent cells in SLOs, which was directly associated with impaired immune surveillance and reduced immune response efficiency [77]. Furthermore, our study confirmed that the expression levels of p16^INK4a^ and p21^Waf1/Cip1^ in SLOs increased in tandem with age, complementing previous findings that indicated these markers may not exhibit simultaneous increases across different cell populations in various organs [78–80], thereby providing supportive data for aging-related studies in SLOs. Consistent with earlier research [76], we observed a decrease in the proportion of CD4^+^ T cells and an increase in the proportion of CD8^+^ T cells with age in the LNs and spleen, while the opposite pattern was observed in the PPs, suggesting organ-specific immune alterations. The gut microbiota plays a pivotal role in the development and functional maturation of the gut immune system, including the GALT such as PPs [81]. In this study, we also investigated the changes in gut microbiota during aging and its relationship with PPs. Our findings indicate that bacterial species, such as *Muribaculum*, which increased, and *Faecalibaculum*, which decreased in the aged mice, were significantly correlated with alterations in cell subpopulations within the PPs. In order to reveal the relationship between them, our study found that aged donor-derived gut microbiota accelerated the aging process of recipient mice with PPs through FMT experiments. The findings confirmed a correlation between aging gut microbiota and the decline of immune function in PPs. However, further experiments, such as bacterial colonisation studies, are necessary to clarify the mechanisms by which gut microbiota influence immunosenescence. Nonetheless, our study provides preliminary experimental evidence supporting the establishment of the gut microbiota-PPs axis. Overall, our findings indicate that the aging process is associated with alterations in spatial distribution, accumulation of senescent cells, and remodelling of immune cell subpopulations with tissue specificity in SLOs. Notably, the gut microbiota emerged as a significant regulator of these changes in PPs. By resolving the immune cell subpopulations, this study identified a strong association with several senescence-related signalling pathways, suggesting that these subpopulations may serve as predictive biomarkers for immunosenescence.

Stromal cells within SLOs exhibit alterations in both quantity and spatial distribution as a consequence of aging, which may affect the structural integrity of follicles and the localisation of macrophages [20, 21]. These stromal cells play a pivotal role in guiding lymphocytes to their respective niches, ensuring effective immune responses [20, 21]. While the stromal cells of primary lymphoid organs have been extensively investigated: evidencing that aging in bone marrow stromal cells can lead to bone loss [82, 83], and that changes in thymic stromal cells are associated with reduced T cell production [84, 85], research focusing on SLO stromal cells remains comparatively limited. Recent investigations have identified two unique statuses of thymic epithelial cells within thymic stromal cells that are linked to tissue degeneration and contribute to the thymic aging [86]. In SLOs, the aging process predominantly impacts the spatial organisation of stromal cells and the expression of chemokines, especially within LNs and the spleen [15, 87]. Concerning age-related changes in FRCs, morphological assessments indicate a reduction in the area occupied by FDCs in LNs with advancing age, while MRCs in the spleen exhibit significant structural disruptions [24, 88]. Our study confirmed these alterations, finding that MRCs become scattered in aged LNs and spleen, likely disrupting the architecture of medullary sinuses and impairing antigen recognition efficiency. This disordered distribution of MRCs affects macrophage localisation, diminishing their clearance capabilities and promoting inflammation [89]. We propose a macrophage-stromal cell circuit in SLOs, where senescent stromal cells alter macrophage distribution through chemokines, and disordered macrophages disrupt the local immune microenvironment, impairing immune homeostasis and contributing to systemic aging [90, 91]. Additionally, we observed reduced FDC areas in LNs, spleen, and PPs, which may lead to decreased antigen capture and presentation [24, 48]. Prados A et al. demonstrated that stromal cells in PPs exhibit a similar localised distribution and chemokine expression pattern as those in LNs and the spleen [92]. We hypothesise that stromal cells in PPs experience similar stressors and alterations during aging, potentially influencing immune cell behaviour through cytokine release and pathogen transport. Recent studies have characterised stromal cell populations in young SLOs by scRNA-seq techniques [92–94], and we anticipate that future research will facilitate a comprehensive analysis of senescent stromal cell populations. In addition, we have demonstrated the accumulation of senescent cells within the stromal cell population. In recent years, an increasing number of studies have indicated that age-related alterations in stromal cells impact vaccination responses [95, 96]. Our study provides novel experimental data supporting the notion of targeting stromal cells therapeutically to enhance immune responses, thereby broadening the scope of intervention research within the field of immunology related to aging.

Morphological alterations serve as prominent indicators for assessing the aging process. Thymic atrophy is widely acknowledged as a key characteristic of immunosenescence [68]. Additionally, LNs and the spleen experience various degenerative changes with advancing age, including atrophy, the accumulation of adipose tissue, and differing degrees of fibrosis [88, 97]. However, these changes have not been sufficiently explored in PPs. Consistent with previous studies, our findings revealed that both LNs and the spleen exhibited size atrophy and mass loss, alongside periosteal thickening, increased fibrosis, and increased stiffness, which may further impair tissue functionality by limiting normal cellular interactions and nutrient transport [18, 98]. Histopathological analyses also indicated a reduction in immune function within both LNs and the spleen. Notably, our study observed an increase in pigmentation, likely attributable to the accumulation of lipofuscin or hemosiderin, which may be associated with oxidative stress and cellular damage. Similarly, PPs showed increased atrophy and fibrosis, although senescent PPs also exhibited mass gain. These age-related structural changes suggest degeneration of SLOs, which may impair immune functions and increase susceptibility to infections and systemic immune aging. Targeted intervention within the SLO microenvironment, cell-based therapies aimed at reconstructing tissue structure or targeting stromal cells to regulate the tissue microenvironment [99] may be potential therapeutic ideas.

In addition, the aging-associated chronic low-grade inflammatory state and its concomitant elevated expression of pro-inflammatory factors, such as IL-6 and IFN-γ, may also differentially influence the survival, proliferation, and retention of immune cells [100]. Recent investigations have indicated that inflammation can trigger an anti-inflammatory response to counteract the age-related pro-inflammatory microenvironment [101]. However, if the inflammatory response does not resolve in a timely manner, persistent chronic inflammation can expedite the aging of immune cells, resulting in diminished immune surveillance. This creates a detrimental cycle of chronic inflammation and immunosenescence, which may ultimately contribute to the development of age-related diseases [102]. There is an urgent need for future research to thoroughly examine the bidirectional regulatory mechanisms of inflammation across various pathological stages, thereby providing a theoretical foundation for the formulation of precise intervention strategies targeting the inflammation-immune senescence axis.

The accumulation of senescent cells is a significant feature of organismal aging, and several studies have demonstrated that the elimination of these cells can enhance tissue function and extend lifespan [103–105]. Recently, senolytics have emerged as promising agents in anti-aging therapies [106, 107], and the accurate identification of senescent cell subpopulations within specific tissues has become crucial for the development of targeted intervention strategies. In this study, we conducted a systematic analysis of age-related changes in cellular and tissue structures within SLOs, revealing the accumulation of senescence in both immune cell subpopulations and stromal cell populations. This finding highlights important cellular targets for addressing SLO aging. A comprehensive investigation into the molecular mechanisms by which these senescent cell subpopulations contribute to immunosenescence is necessary to clarify potential therapeutic directions. Meanwhile, it was found that the gut microbiota plays an important regulatory role in the process of immunosenescence, suggesting that modulation of the gut microbiota through dietary interventions and faecal transplantation may improve immunosenescence [108]. While this study offers new insights into the characterisation of the aging landscape and intervention strategies within SLOs, it is important to acknowledge certain limitations. The current conclusions are mainly based on mouse models, and their translation to humans requires further verification due to interspecies differences. Furthermore, the dynamic interactions between senescent cells and the regulatory network of the gut microbiota-immunity axis also need to be further elucidated. Future research should enhance our understanding of the mechanisms underlying human immunosenescence and facilitate the translation of foundational findings into effective prevention and treatment strategies for immunosenescence through clinical studies.

## Materials And Methods

### Animals

All animal experiments were approved by the Institutional Animal Ethics Committee of Shenzhen Top Biotech Co., Ltd. (Approval No.: TOPGM-IACUC-2024-0011) and performed in accordance with the institutional guidelines for animal care and use. Mice were kept under specific pathogen-free conditions at Shenzhen Top Biotech Co., Ltd. (Shenzhen, China), with an ambient temperature of 23 ± 2 °C, 40% humidity, and a 12 h light-dark cycle. Wild-type (WT) C57BL/6 J male mice were sacrificed at either 2–3 months or 18–20 months of age following an 8 h fasting period [14, 51, 109]. Post-euthanasia, tissue samples were collected as previously described for further analyses [110, 111]. For flow cytometry, immunofluorescence staining and faecal collection, each age group consisted of 5 mice. For histology and immunohistochemical staining, each age group consisted of 6 mice.

### Single-cell RNA sequencing (scRNA-seq) data analysis

Raw scRNA-seq data for LNs were obtained from the Gene Expression Archive (GSA accession number CRA004687) at the Genome Sequence Archive, BIG Data Center, Beijing Institute of Genomics (BIG, https://bigd.big.ac.cn/gsa/), Chinese Academy of Sciences. Spleen scRNA-seq data were retrieved as raw reads from the Gene Expression Omnibus (GEO, http://www.ncbi.nlm.nih.gov/geo) under accession number GSE132042. Differentially expressed gene sets between young and aged LNs and spleen samples in CRA004687 and GSE132042 were identified using the GCBI online platform. Quality control was performed as described in previous studies [112, 113]. Sequence demultiplexing and barcode mapping were performed using the “cellranger count” command from CellRanger Software Suite (version 3.1.0). The Seurat package was utilised to generate the single-cell expression matrix, followed by data filtering, normalisation, dimensionality reduction, clustering, and differential gene expression analysis. The number of genes detected, the proportion of mitochondrial genes and ribosomal genes were used to filter out low-quality cells. After filtering, we obtained 22,203 high-quality cells from lymph nodes (12,294 cells from the young group and 9909 cells from the old) and 35,718 cells from the spleen (10,123 cells from the young group and 25,595 cells from the old). DEGs across conditions were found using the FindMarkers function and the default Wilcoxon rank test. Genes were sorted by absolute log2 fold-change (log2FC). Genes having *P*-values > 0.05 (adjusted for multiple comparisons), min.pct < 0.05, or log2FC < 0.1 were eliminated. The clusterProfiler R package was used to perform GO enrichment analysis of DEGs [114]. The marker genes in each cluster were compared to a list of classical reference marker genes, and the clusters were assigned to the appropriate cell types based on the degree of match. The clusterProfiler R tool was used to complete GO enrichment analysis of DEGs. GO terms were deemed substantially enriched by DEGs if their adjusted *P* value was less than 0.05.

### Flow cytometry analysis

Single-cell suspensions were prepared as described in previous studies [115, 116] and resuspended in PBS with 2% FBS. Cells were incubated with Fc receptor-blocking antibodies and subsequently stained with a surface antibody cocktail in stain buffer (BD) following the instructions. Antibodies used included CD45 (Cat# 103107 BioLegend), CD3 (Cat# 100218 BioLegend), CD4 (Cat# 100407 BioLegend; Cat# 740105 BD Bioscience), CD8a (Cat# 56-0081-82 Thermo Fisher Scientific), CD44 (Cat# 103011 BioLegend), CD62L (Cat# 48-0621-82 Thermo Fisher Scientific), CXCR3 (Cat# 752155 BD Bioscience), CD154 (Cat# 740480 BD Bioscience), CD19 (Cat# 115543 BioLegend; Cat# 17-0193-82 Thermo Fisher Scientific), NK1.1 (Cat# 11-5941-82 Thermo Fisher Scientific), IgD (Cat# 48-5993-82 Thermo Fisher Scientific), CD27 (Cat# 25-0271-82 Thermo Fisher Scientific), TIM-3 (Cat# 568797 BD Bioscience), CXCR5 (Cat# 560617 BD Bioscience), PD-1 (Cat# 748268 BD Bioscience), CD31 (Cat# 102407 BioLegend) and Podoplanin (Cat# 62-5381-82 Thermo Fisher Scientific). In specific experiments, cells were fixed and permeabilised following the instructions, followed by intranuclear or intracytoplasmic staining with antibodies against Granzyme B (Cat# 396437 BioLegend), Foxp3 (Cat# 562996 BD Bioscience), p16 (1:50, Cat# sc-1661 Santa Cruz), or p21 (1:50, Cat# sc-6246 Santa Cruz). Data acquisition was performed using FACSDiva V7.0 software (BD) and analysed with FlowJo V10.6 software (Tree Star).

### Immunofluorescence (IF) staining

For co-staining of p21 and B220/CD3, frozen LNs and spleen tissues were utilized. The MFI of p21 within defined regions was quantified using Fiji software. Prior to MFI calculation, the freehand selection tool was employed to delineate B cell regions (B220-positive) and T cell regions (CD3-positive).

To identify regions corresponding to MRCs, FDCs, B cells, T cells, LEV, macrophages, M cells and epithelial cells, immunofluorescent staining was performed using antibodies against Podoplanin (Cat# sc-376962 Santa Cruz), MAdCAM-1 (Cat# sc-365934 Santa Cruz), ER-TR7 (Cat# sc-73355 Santa Cruz), B220 (Cat# 553087 BD Bioscience), CD3 (Cat# 17-0031-82 Thermo Fisher Scientific), CD21/35 (Cat# 123411 BioLegend), LYVE-1 (Cat# sc-65647 Santa Cruz), F4/80 (Cat# 123131 BioLegend), CD169 (Cat# 142404 BioLegend), CCL20 (Cat# 26527-1-AP Proteintech), GP2 (Cat# D278-3 MBL), and Ep-CAM (Cat# 118211 BioLegend).

Tissue samples were fixed with 4% neutral-buffered paraformaldehyde (Biosharp) for 4–24 h, and then dehydrated in 30% sucrose. After embedding in optimal cutting temperature compound, tissue samples were sectioned into frozen slices, followed by incubation in PBS containing 0.5% Triton X-100 (Diamond) for 15 min at 4 °C. After permeabilisation, samples were blocked with 10% BSA for 30 min. Subsequently, the cells were incubated with primary antibodies overnight at 4 °C. After washing sufficiently, the cells were incubated with secondary antibodies for 60 min at 37 °C. Slides were then washed and mounted with ProLong Gold containing DAPI (Life Technologies) and visualised using a Zeiss 880 Laser Scanning Confocal Microscope with Airyscan (ZEISS, Germany) and an Andor Dragonfly 202 Imaging System high-speed spinning disk confocal microscope.

### Immunohistochemical (IHC) staining

IHC staining was applied to formalin-fixed, paraffin embedded sections. Slides were deparaffinised by heating at 65 °C for 180 min, followed by two 5-min washes in xylene. Tissues were rehydrated through sequential immersion in decreasing concentrations of ethanol, from 100% ethanol to 100% water. 3% hydrogen peroxide in methanol was used to inhibit the endogenous peroxidase activity. After PBS washing, the sections were blocked in blocking buffer in a humidified chamber for 30 min at room temperature. Primary antibodies (α-SMA, 1:500 or collagen-I, 1:100) were applied for incubation overnight at 4 °C. Following PBS washes, secondary antibodies (Abcam) were applied at a 1:2000 dilution for 30 min. Following another PBS wash, DAB (Dako) was used for staining development, and slides were briefly rinsed in water. Haematoxylin counterstaining was performed, and slides were mounted. Imaging was conducted using a microscope and was analysed with ImageJ software (version 1.46).

### Hematoxylin and eosin (HE) staining

After sacrifice, mouse tissue samples were perfused with 4% paraformaldehyde before being embedded in paraffin. Tissue slices were deparaffinised and rehydrated, then stained for 5 min with hematoxylin solution (ZSGB-BIO, China). They were then dipped 5 times in 1% acid ethanol (1% HCl in 75% ethanol) and rinsed with distilled water. Sections were then stained with eosin solution (ZSGB-BIO, China) for 3 min, dehydrated using graded alcohol, and cleared in xylene. Mounted slides were viewed and imaged using a microscope.

### Atomic force microscopy (AFM)

Mouse tissues were embedded in optimal cutting temperature compound and sectioned into frozen slices. AFM was utilised to record the height and phase signals of LN and spleen tissue sections. Images were analysed using NanoScope version 1.4 to evaluate optical, structural, and electrical properties.

### Faecal collection, 16S rRNA gene sequencing, and gut microbiota data analysis

Fresh faecal pellets were collected from individual mice under sterile conditions. To minimise environmental contamination, pellets were immediately transferred to cryotubes using sterilised forceps within 5 min of defecation. Samples were stored at −80 °C until DNA extraction. All shipments to sequencing facilities were conducted on dry ice. Faecal DNA extraction and 16S rRNA sequencing were performed by Novogene Co Ltd. Briefly, faecal DNA was extracted using the Omega Mag-Bind Stool DNA kit (Omega Bio-Tek, Norcross, GA, USA), and assessed for quality via 1.2% agarose gel electrophoresis. The V4 region of the 16S rRNA gene was amplified using bar-coded primers through polymerase chain reaction (PCR). The resulting PCR products were sequenced using a Mi-Seq Illumina sequencer. Bacterial sequence data were processed using the DADA2 pipeline within the Quantitative Insights into Microbial Ecology2 (QIIME2) software (v.2019.4). Spearman’s rank correlation coefficient was calculated to evaluate the relationship between gut microbial species and host Peyer’s patch cell types.

### Faecal microbiota transplantation (FMT) experiment

Faeces were collected from young or aged donor mice. Fresh specimens underwent saline dilution (40 mg/mL), homogenisation, and filtration through a 0.25 mm stainless steel sieve. The bacterial load was normalised by quantifying total protein concentration in faecal filtrates via BCA assay. The processed suspensions were supplemented with 10% sterile glycerol, aliquoted, and stored at −80 °C. SPF C57BL/6 J recipient mice received 200 μL of faecal mixture via oral gavage twice weekly for 6 weeks. At 24 h post-final transplantation, recipients were euthanised for PP collection.

### Statistical analysis

All data were obtained from a minimum of five independent experiments and expressed as mean ± standard deviation (SD). Sample sizes for each experiment were specified in the corresponding figure legends. Comparisons between two groups were conducted using unpaired *t*-tests. Prism software (GraphPad) was used for data analysis. A two-sided *P*-value below 0.05 was considered statistically significant.

## Supplementary information

Comprehensive Characterisation of Age-Related Changes in Cell Subpopulations and Tissue Structural Properties in Secondary Lymphoid Organs

## Data Availability

Data supporting the findings of this study are available from the corresponding authors upon reasonable request.
