# Supplementary material for: Comprehensive characterisation of age-related changes in cell subpopulations and tissue structural properties in secondary lymphoid organs
Source: Cell Death Dis. 2025 Oct 6;16(1):679. doi: 10.1038/s41419-025-08007-y (PMC12501032; doi:10.1038/s41419-025-08007-y)
Supplement: Supplementary file 1 — Comprehensive Characterisation of Age-Related Changes in Cell Subpopulations and Tissue Structural Properties in Secondary Lymphoid Organs [file 41419_2025_8007_MOESM1_ESM.pdf]

Supporting Information

Comprehensive Characterization of Age-Related Changes in Cell Subpopulations and Tissue Structural Properties in Secondary Lymphoid Organs

Yuxin Deng, Xin He, Juzheng Peng, Yuxi Pan, Yusheng Luo, Yueheng Ruan, Jianfeng Hou, Bangxue Jiang, Xiangyu Li, Xiaomei Liang, Jiayuan Huang[#], Jiancheng Wang[#]

The PDF file includes:

Fig. S1 to 6

Table S1

Fig. S1

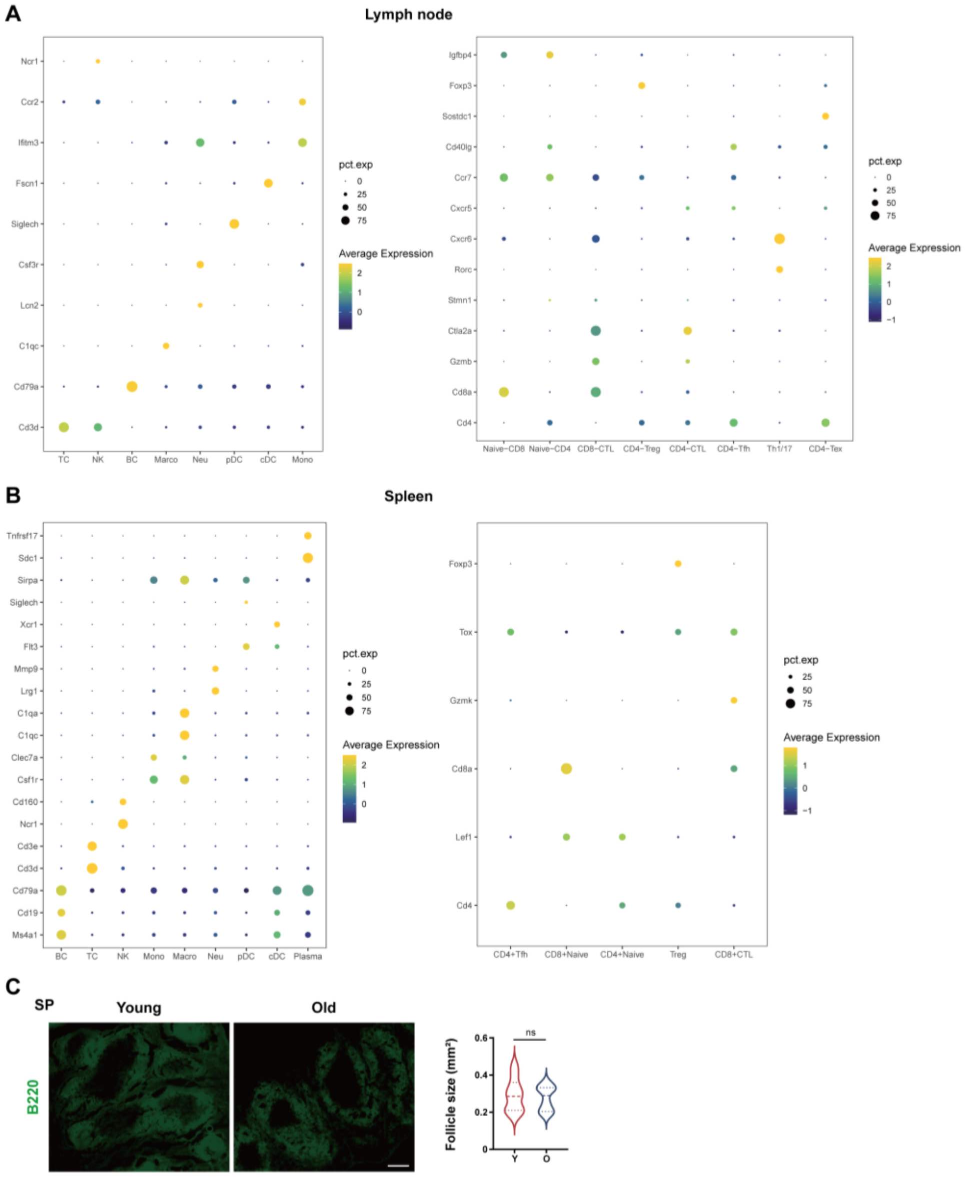

(A) The heatmap illustrating the average expression levels of key genes across different cell clusters and T cell subsets in young and old LNs. (B) The heatmap illustrating the average expression levels of key genes across different cell clusters and T cell subsets in young and old spleen. (C) Histological sections of spleen with immunostaining for B cells (B220, green) and measurement of the area of B220^{+} B cell follicles in young and old mice. Scale bars represent 200 μm (n = 5 mice/group). *P*-values were calculated between two groups using an unpaired *t*-test. ns, not significant; **P*<0.05; ***P*<0.01; ****P*<0.001.

Fig. S2

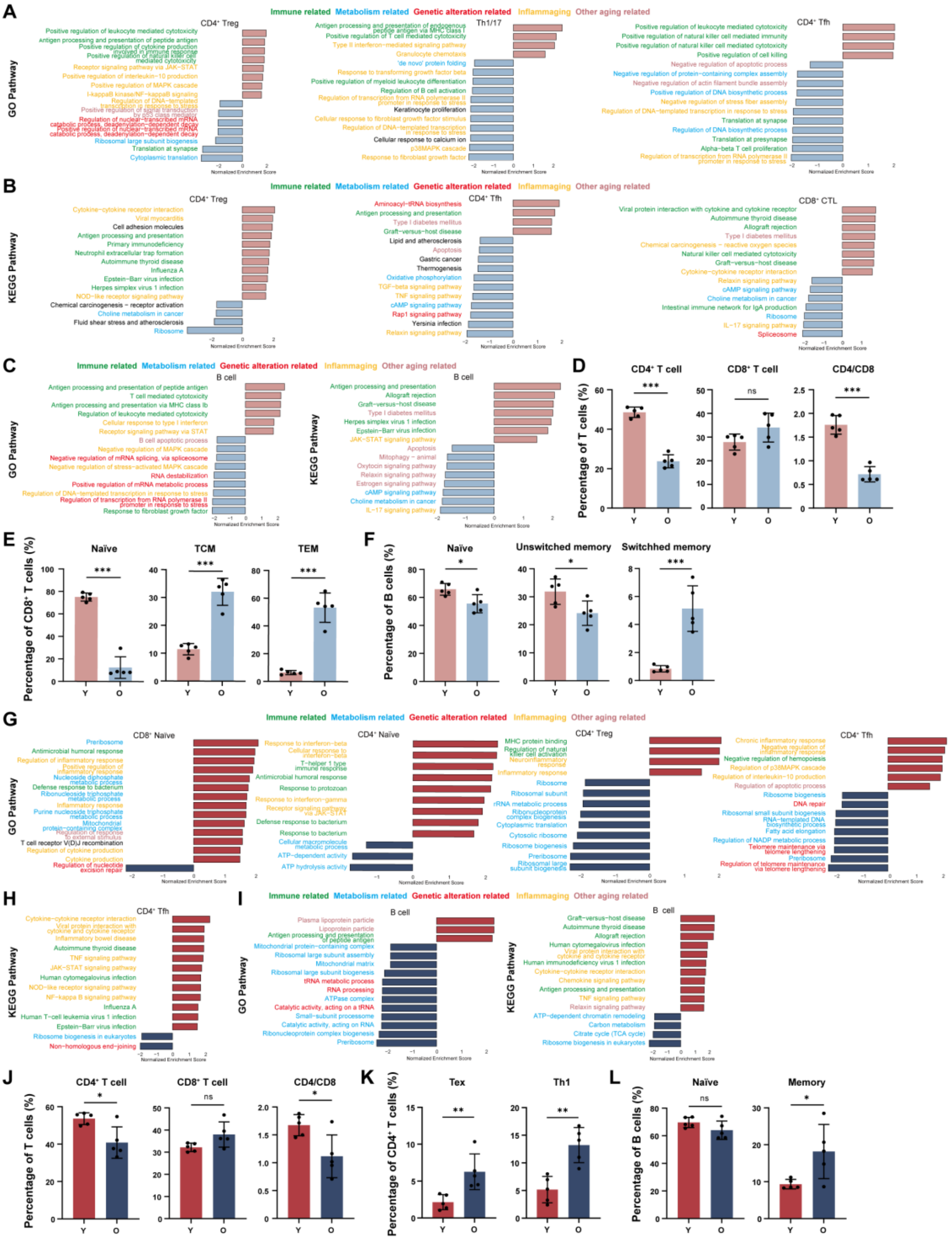

(A-B) NES of enrichment for GO biological process terms and KEGG terms with an age-related change. Only top 15 GO or KEGG terms with *P*-value < 0.05 were listed for all T subsets in young and old LNs. (C) NES of enrichment for GO biological process terms and KEGG terms with an age-related change. Only top 15 GO or KEGG terms with *P*-value < 0.05 were listed for B cells in young and old LNs. (D) Flow cytometric analysis of $CD4^{+}$ T cells and $CD8^{+}$ T cells was performed. The

percentage representation of the populations was shown in young and old LNs (n = 5 mice/group). (E) Flow cytometric analysis of $CD8^+$ naïve T cells, TCM cells and TEM cells was performed. The percentage of the populations was shown in young and old LNs (n = 5 mice/group). (F) Flow cytometric analysis of naïve B cells ($CD19^+$, IgD^+, $CD27^-$), unswitched memory cell ($CD19^+$, IgD^+, $CD27^+$) and switched memory cell ($CD19^+$, IgD^-, $CD27^+$) was performed. The percentage representation of the populations was shown in young and old LNs (n = 5 mice/group). (G-H) NES of enrichment for GO biological process terms and KEGG terms with an age-related change. Only top 15 GO or KEGG terms with P-value < 0.05 were listed for each major T cell subset in young and old spleen. (I) NES of enrichment for GO biological process terms and KEGG terms with an age-related change. Only top 15 GO or KEGG terms with P-value < 0.05 were listed for B cells in young and old spleen. (J) Flow cytometric analysis of $CD4^+$ T cells and $CD8^+$ T cells was performed. The percentage of the populations was shown in young and old spleen (n = 5 mice/group). (K) Flow cytometric analysis of $CD4^+$ Tex cells and $CD4^+$ Th1 cells was performed. The percentage of the populations was shown in young and old spleen (n = 5 mice/group). (L) Flow cytometric analysis of naïve B cells and memory B cells was performed. The percentage of the populations was shown in young and old spleen (n = 5 mice/group). P-values were calculated between two groups using an unpaired t-test. ns, not significant; *$P<0.05$; **$P<0.01$; ***$P<0.001$.

Fig. S3

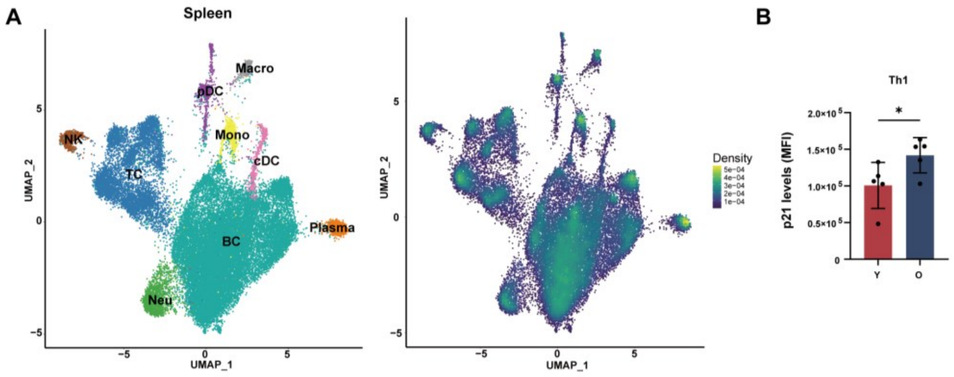

(A) *Left Panel*: UMAP plot illustrating subclusters of cell subsets in young and old spleen; *Right Panel*: Density distribution plot of aging scores. (B) Flow cytometric analysis of p21 levels in Th1 cells populations in young and old spleen (n = 5 mice/group). *P*-values were calculated between two groups using an unpaired *t*-test. ns, not significant; **P*＜0.05; ***P*＜0.01; ****P*<0.001.

Fig. S4

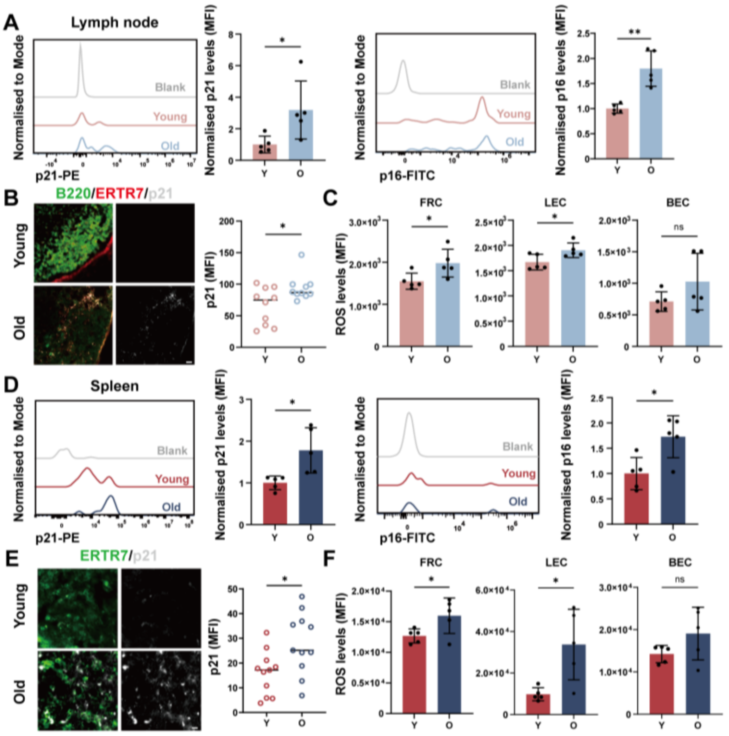

(A) Flow cytometric analysis of p21 and p16 levels in stromal cells ($CD45^-$, $PDPN^+$) in young and old LNs (n = 5 mice/group). (B) Histological sections of LNs with immunostaining for stromal cells (ERTR7, red) and measurement of p21 MFI of $ERTR7^+$ stromal cell area in young and old LNs. Scale bars represent 50 μm (n = 5 mice/group). (C) Flow cytometric analysis of ROS levels in FRCs ($PDPN^+$, $CD31^-$), LECs ($PDPN^+$, $CD31^+$) and BECs ($PDPN^-$, $CD31^+$) in young and old LNs (n = 5 mice/group). (D) Flow cytometric analysis of p16 and p21 levels in stromal cells ($CD45^-PDPN^+$) in young and old spleen (n = 5 mice/group). (E) Histological sections of spleen with immunostaining for stromal cells (ERTR7, green) and measurement of p21 MFI of $ERTR7^+$ stromal cell area in young and old spleen. Scale bars represent 10 μm (n = 5 mice/group). (F) Flow cytometric analysis of ROS levels in FRCs ($PDPN^+$, $CD31^-$), LECs ($PDPN^+$, $CD31^+$) and BECs ($PDPN^-$, $CD31^+$) in young and old spleen (n = 5 mice/group). *P*-values were calculated between two groups using an unpaired *t*-test. ns, not significant; $*P<0.05$; $**P<0.01$; $***P<0.001$.

Fig. S5

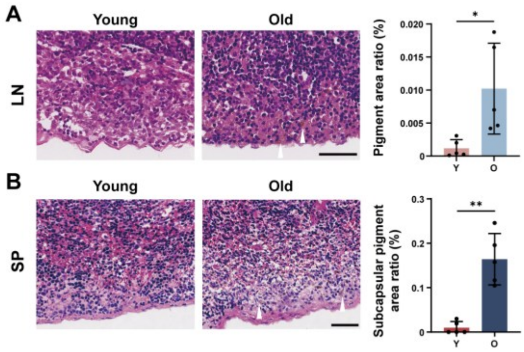

(A) Representative images of LN tissue sections after HE staining and measurement of pigment area within the visual field in young and old mice. Scale bars represent 50 μm (n = 5 mice/group). (B) Representative images of spleen tissue sections after HE staining and measurement of subcapsular pigment area within the visual field in young and old mice. Scale bars represent 50 μm (n = 5 mice/group). *P*-values were calculated between two groups using an unpaired *t*-test. ns, not significant; *$P<0.05$; **$P<0.01$; ***$P<0.001$.

Fig. S6

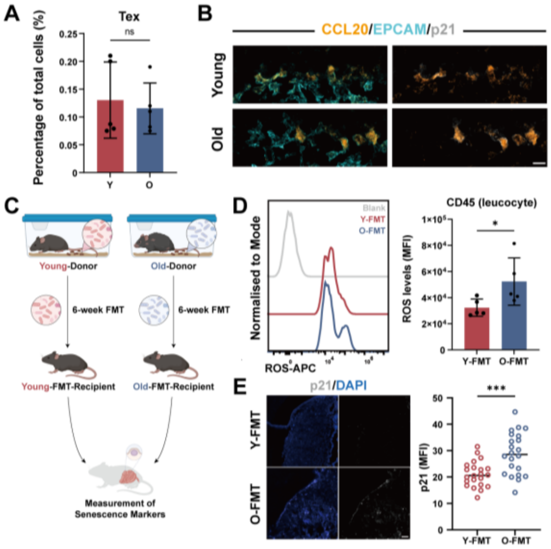

(A) Flow cytometric analysis of $CD4^+$ Tex cells was performed. The percentage of the populations was shown in young and old PPs (n = 5 mice/group). (B) Histological sections of intestinal PPs with immunostaining for mature M cells (CCL20, bright orange) and epithelial cells (EPCAM, soft cyan). Scale bars represent 50 μm (n = 5 mice/group). (C) Illustration of faecal microbiota transplantation (FMT) experiment. (D) Flow cytometric analysis of ROS levels in $CD45^+$ leucocyte populations in PPs from young-FMT recipients and old-FMT recipients (n = 5 mice/group). (E) Immunostaining of PP histological sections and measurement of p21 MFI from young-FMT recipients and old-FMT recipients. Scale bars represent 50 μm (n = 5 mice/group). *P*-values were calculated between two groups using an unpaired *t*-test. ns, not significant; *$P<0.05$; **$P<0.01$; ***$P<0.001$.

Table S1. Key Resources

ANTIBODIES	SOURCE	IDENTIFIER
Waf1/Cip1/CDKN1A p21 antibody (F-5)	Santa Cruz Biotechnology	Cat# sc-6246; RRID: AB_628073
CDKN2A/p16 antibody (F-12)	Santa Cruz Biotechnology	Cat# sc-1661; RRID: AB_628067
LYVE-1 antibody (E9VA4)	Santa Cruz Biotechnology	Cat# sc-65647; RRID: AB_1123635
podoplanin antibody (F-3)	Santa Cruz Biotechnology	Cat# sc-376962
MAdCAM-1 antibody (H-3)	Santa Cruz Biotechnology	Cat# sc-365934; RRID: AB_10916705
Fibroblast Marker antibody (ER-TR7)	Santa Cruz Biotechnology	Cat# sc-73355; RRID: AB_1122890
APC anti-mouse CD21/CD35 (CR2/CR1) Antibody	BioLegend	Cat# 123411; RRID: AB_940395
FITC anti-mouse CD45 Antibody	BioLegend	Cat# 103107; RRID: AB_312972
PerCP/Cyanine5.5 anti-mouse CD3 Antibody	BioLegend	Cat# 100218; RRID: AB_1595492
Brilliant Violet 785™ anti-mouse CD19 Antibody	BioLegend	Cat# 115543; RRID: AB_11218994
PE anti-mouse CD4 Antibody	BioLegend	Cat# 100407; RRID: AB_312692
APC anti-mouse/human CD44 Antibody	BioLegend	Cat# 103011; RRID: AB_312962
PE anti-mouse CD169 (Siglec-1) Antibody	BioLegend	Cat# 142404; RRID: AB_10915697
PE anti-mouse CD31 Antibody	BioLegend	Cat# 102407; RRID: AB_312902
Brilliant Violet 421™ anti-mouse F4/80 Antibody	BioLegend	Cat# 123131; RRID: AB_10901171
Brilliant Violet 785™ anti-human/mouse Granzyme B Recombinant Antibody	BioLegend	Cat# 396437; RRID: AB_3106140
Alexa Fluor® 647 anti-mouse CD326 (Ep-CAM) Antibody	BioLegend	Cat# 118211; RRID: AB_1134104
CCL20/MIP-3 alpha Polyclonal antibody	Proteintech	Cat# 26527-1-AP; RRID: AB_2880543
CD3e Monoclonal Antibody (145-2C11) APC	Thermo Fisher Scientific	Cat# 17-0031-82; RRID: AB_469315
CD19 Monoclonal Antibody (eBio1D3 (1D3)) APC	Thermo Fisher Scientific	Cat# 17-0193-82; RRID: AB_1659676
CD8a Monoclonal Antibody (53-6.7) Alexa Fluor™ 700	Thermo Fisher Scientific	Cat# 56-0081-82; RRID: AB_494005
CD62L (L-Selectin) Monoclonal Antibody (MEL-14) eFluor™ 450	Thermo Fisher Scientific	Cat# 48-0621-82; RRID: AB_1963590
CD27 Monoclonal Antibody (LG.7F9) PE-Cyanine7	Thermo Fisher Scientific	Cat# 25-0271-82; RRID: AB_1724035
IgD Monoclonal Antibody (11-26c (11-26)) eFluor™ 450	Thermo Fisher Scientific	Cat# 48-5993-82; RRID: AB_1272202
Podoplanin Monoclonal Antibody (eBio8.1.1(8.1.1)), Super Bright™ 436	Thermo Fisher Scientific	Cat# 62-5381-82; RRID: AB_2744800
NK1.1 Monoclonal Antibody (PK136), FITC	Thermo Fisher Scientific	Cat# 11-5941-82; RRID: AB_465318
FITC Rat Anti-Mouse CD45R/B220	BD Bioscience	Cat# 553087; RRID: AB_394617
BV510 Rat Anti-Mouse CD4	BD Bioscience	Cat# 740105; RRID: AB_2739863
R718 Hamster Anti-Mouse CD183 (CXCR3)	BD Bioscience	Cat# 752155; RRID: AB_2917263
Alexa Fluor™ 647 Rat Anti-Mouse CD366 (TIM-3)	BD Bioscience	Cat# 568797
PE-Cy™7 Rat Anti-Mouse CD185 (CXCR5)	BD Bioscience	Cat# 560617; RRID: AB_1727521
BV650 Hamster Anti-Mouse CD154	BD Bioscience	Cat# 740480; RRID: AB_2740205
Ms CD279 (PD-1) BV421 RMP1	BD Bioscience	Cat# 748268; RRID: AB_2872696
BV421 Rat Anti-Mouse Foxp3	BD Bioscience	Cat# 562996; RRID: AB_2737940
Anti-GP2 (Glycoprotein 2) (Mouse) mAb	MBL Life Sciences	Cat# D278-3
